# Unveiling the silent suffering: Examining the complexities of disclosure and concealment strategies in women living with obstetric fistula

**DOI:** 10.1016/j.heliyon.2024.e38824

**Published:** 2024-10-02

**Authors:** Sandra Freda Wood, Ebenezer Ato Kwamena Aidoo, Kweku Ewusie Orleans Lindsay, Jessica Afful Tuleassi, Frank Nukunu, Cindy Afoakwa-Acheampong

**Affiliations:** a Hugh Downs School of Human Communication, Arizona State University, Tempe, USA; b Communication Studies Department, University of Iowa, Iowa City, USA; c Nursing and Midwifery Training College, Cape Coast, Ghana; d Hubbard School of Journalism and Mass Communication, University of Minnesota, Twin Cities USA; e Brian Lamb School of Communication, Purdue University, West Lafayette USA

**Keywords:** Communication privacy management (CPM), Disclosure/non-disclosure, Ghana/Africa, Obstetric fistula, Strategies

## Abstract

Obstetric Fistula (OF), one of the tragic birth injuries in developing countries, overwhelms women living with this condition with multifaceted effects. Although concealing has been justified in some studies, revealing is also encouraged. Such uncertainty leaves women in a loop of tension in managing the disclosure or non-disclosure of their diagnosis. They resort to several strategies to manage their private information. Using Communication Privacy Management (CPM) Theory, this study documents the disclosure or non-disclosure strategies that women living with OF adopt to manage their diagnosis information. Data was derived from semi-structured interviews conducted with 19 women either living with OF presently or have lived with it before. The thematic analysis yielded categories of who, when, what, and how. These categories were further subdivided into nine specific practices or strategies for revealing. However, the categories under the non-disclosure focused on only who and how, where four themes emerged. This study has both practical and theoretical significance by identifying disclosure and non-disclosure intervention strategies useful for providing relief for women diagnosed with OF.

## 1 Introduction

### 1.1 “In an unequal world, these women are the most unequal among unequal” [1]

Obstetric Fistula (OF) is one of the most severe and tragic childbirth injuries which primarily affects women in impoverished nations [2]. Notably, in Sub-Saharan Africa and Asia, an estimate of more than half a million women or girls have been estimated to suffer from OF with the number increasing annually [1,3,4]. According to Ghana Health Services [GHS] (2015), OF reported cases in Ghana are about one thousand five hundred, with a yearly incidence increase of 200 cases and only 40 % of these cases are fortunate enough to get “repaired” [5]. Although it is a preventable condition, its existence shows the gross inequities in health care systems in developing countries.

Obstetric fistula (OF) is an improper connection between the genital tract and the urinary tract, or the genital tract and the digestive tract, brought on by protracted obstructed labor [6,7]. There are many kinds of OFs such as vesicovaginal fistulas (VVF), which occur between the bladder and vagina, rectovaginal fistulas (RVF), which occur between the rectum and the vagina, ureterovaginal fistulas (UVF), which occur between the urethra and the vagina, and ureterovaginal fistulas, which occur between the ureters and the vagina [1,8]. This condition is often a result of several factors including poverty, poor access to healthcare, lack of logistics/equipment at healthcare centers, sociocultural factors, discrimination against women, early marriage, and lack of qualified birth attendants [9]. Consequently, women or girls in this condition experience uncontrollable leakage of urine or feces, or sometimes both feces and urine, which leaves them with severe physical effects. Intensified effects from OF cause mothers to lose their babies, especially during delivery as well [10].

There are multiple dire effects associated with OF. Women living with this condition experience poor treatment in their relationships which makes them prone to stigmatization, isolation, divorce or separation from their partners, suicidal ideation, despair, psychological trauma, feeling abandoned, and economically unstable [11,12]. Even though there is a 70–90 % success rate for curing OF, there is a lack of skilled surgeons to treat these women [13]. Apart from these effects on the self, Jarvis et al. [14] established that the families of these women living with OF also experience stigma even as caregivers. Some of these women are left to practically live with the condition for several years with no assurance of getting treatment. For those who are fortunate and get treated, they also face challenges in reintegrating themselves into society.

The intensity of's effects result in most women losing their babies during delivery The first argument by Ghana Health Service contends that these women are socialized mostly by their families to keep their diagnosis “private” to avoid all the potential risks associated with stigmatization [5]. Other studies have supported this view by establishing that revealing private information can intensify avoidance, stigmatization and worsen the state of the one disclosing [[15], [16], [17]]. Thus, a concern for some degree of privacy is justified. The second argument holds that healthcare experts encourage disclosure to enable these women gain some support from family, friends, and the community [18]. Empirically, Pennebaker et al. [19] affirmed that between 70 and 89 % of their respondents endorsed disclosure of emotional because disclosure brings relief and increases intimacy. Therefore, the refusal to disclose emotional events has been associated with lower psychological well-being and poorer health [19]. The dilemma associated with disclosure and non-disclosure of diagnosis has left most women with the condition so distraught that they stay hidden, isolated and struggle with how to manage the diagnosis information [9]. Recognizing this pull-push tension between disclosure and non-disclosure emphasizes the nuances related to the outcome of disclosure or concealment. Interestingly, in the Ghanaian context, there is a gap in understanding how this tension of privacy management happens among women living with OF.

Consequently, this research paper seeks to contribute to health and privacy management literature by focusing on OF as a prevalent birth injury in developing countries and examining the disclosure and non-disclosure strategies employed by women living with OF. By utilizing the Communication Privacy Management (CPM) Theory, the study provides insights into the complex dynamics of disclosure decisions, offering practical, and theoretical implications. Additionally, the study expands the theoretical understanding of privacy management and disclosure processes in the context of stigmatized health conditions. Overall, this research brings a non-Western perspective, highlights cultural differences, and presents disclosure and non-disclosure as a process rather than binary opposites, while at the same time, offering a comprehensive range of practices utilized by Ghanaian women with OF.

## 2 Literature review

### 2.1 The concept of disclosure or non-disclosure

Disclosure or revealing and non-disclosure or concealing as part of the terms in this study will be used interchangeably with other synonymous words as noticed in many studies to create variety in word use, although a few studies have shared some differences. Nielson et al. [20] contend that disclosure is a complex process that encompasses the degree of openness in communicating an issue or a condition. In simple terms, Obermeyer et al. [21] explain disclosure as whom to speak to, what to communicate about, the way the communication takes place, when to talk about the condition and the outcome and experiences of the condition. Sharing delicate information in most cases makes a person vulnerable [22,23]. Individuals who receive a diagnosis regarding their health experience psychological and emotional trauma including fear and uncertainty [24]. Studies on individuals’ decision to engage in disclosure/non-disclosure of personal/private health information have stated that there can be both positive and negative outcomes [25,26], though the context of the type of condition also matters [27,28]. Highlighting stigmatized conditions, previous studies have focused on HIV/AIDS [[29], [30], [31]], mental illness [[32], [33], [34]], cancer [35], endometriosis [36], infertility [37], abuse and violence [38,39] which are not unique to the Global South or African context.

Disclosure/non-disclosure communicative practices or strategies in health.

Patients are overwhelmed by the sheer amount of health information they receive from practitioners, which they must make some risk-benefit analysis to evaluate whether sharing or hiding the information will be the best decision. Researchers have established that in most of these situations, patients evaluate the degree of risk to the self, others and relationships [26,40]. These researchers found that when people perceive the outcome of the disclosure to be negative, there is the likelihood of concealing the diagnosis. However, when it is positive, they proceed to disclose the diagnosis other research findings predict and show the kinds of practices and strategies people employ to navigate the complex decision-making process [[41], [42], [43]].

Greene [24], who developed the integrated model of health disclosure decision making, identifies gossip, topic avoidance, concealment, and disclosure as some of the strategies or practices individuals use to manage their private information. In this study, both disclosure and concealment are used as an “active process” rather than a strategy which is similar to Venetis et al.’s [43] adoption of these terms. Similarly, Afifi and Steuber [40] identified six strategies people use to reveal their secret. These were: preparation and rehearsal, directness, third-party, incremental, entrapment, and indirect mediums. Another study investigated strategies or practices that were often adopted by people living with HIV/AIDS and they found eight disclosure practices that were categorized under three main themes. These include mode: face-to-face, non-face to face and third party; context: setting, planning a time, and bringing a companion; content: practicing, incremental disclosure [42]. In recent research by Nielsen et al. [20] about how disclosure affects adults with diabetes self-management, found two main disclosure practices used by their participants. These were open and closed. These studies show how people manage their private information though most of these focused more on disclosure practices than non-disclosure practices which create another gap for this present study to explore.

### 2.2 Communication Privacy Management (CPM) theory

Developed in the field of communication but used in several other fields, Communication Privacy Management Theory (CPM) focuses on understanding how people regulate private information in relation to revealing or concealing [26,44]. Emphasis is placed on the “dialectic tension” one engages in deciding to tell or not tell others about their private information [45]. Dialectic tension is the inherent tensions or contradictions that individuals face in managing their private information within interpersonal relationships [46]. Expanding on this, the theorists explained how individuals negotiate privacy boundaries or rules to regulate the flow of information as they decide to make others co-owners or confidant of their private information [45]. The theory proposes that individuals own their private information and grant others partial ownership when they open a privacy boundary. Individuals use a rules-based management system to regulate the flow of private information to mitigate disclosure risks. CPM therefore contends that individuals’ decisions regarding the disclosure or concealment of private information are influenced by culture, motivation, context, and benefit–risk evaluation [46].

The theory rests on three elements: privacy ownership, privacy control, and privacy turbulence with eight privacy management principles [47], which altogether impacted this present study in several ways. However, relevant to the current study, two out of three elements predominantly guided the analysis of this work. These were the privacy rule characteristics (privacy ownership) and boundaries coordination (privacy control). These two have greater importance for this study because of the scope of understanding the practices involved in the ownership of the information by patients living with OF and how they set boundaries and rules as part of these strategies to manage their diagnosis disclosure or non-disclosure. Thus, these “permeable” and “impermeable” boundaries are created to regulate who they make as confidants, the amount of information, when they share, and how they reveal or conceal.

Several studies have used CPM in different areas or contexts such as family, social media, friendship, social network, workplace, health, and relational issues [47]. In their study about infertility, Bute and Vik [48] acknowledged the danger of reducing disclosure or non-disclosure as a single event without recognition of the complex processes involved in information privacy management. CPM as a theoretical framework has been employed in several studies in understanding how people manage their information privacy boundaries systems. Applying CPM to the Ghanaian cultural context will provide an added perspective due to Ghana's unique beliefs, values, healthcare access, marriage, and family systems, as well as women's status and healthcare in general.

### 2.3 Ghanaian cultural context

This research was conducted in Ghana, with most of the participants coming from the northern and southern parts of Ghana which are known to have higher OF rates [49]. OF prevalence has been traced to low facility delivery specifically in the Northern (37 %) and Central (61 %) regions of the country. Though each region has its differing privileges and development particularly with healthcare there is still enormous overlap. This paper focuses more on the people of the northern part of Ghana to reflect the beliefs, values, customs and traditions of the majority of participants who took part in the study (thirteen out of nineteen). We describe only cultural issues relevant to the findings.

Using Hofstede's culture index, Ghana is said to lean more towards collectivist, average masculine and high-power distance cultures [50]. This means that at the national level, Ghanaians value group identity and have a high regard for hierarchy. This impacts the regional cultures. As culture evolves, it is worth noting that some of these characterizations may not be reflected in some individuals based on several factors such as education, social status, economic power, and others. The marriage system within the northern parts is polygamous [51]. Polygamous marriages mostly give rise to a larger family system that lives together in a compound house with the husband as the “head” [52]. This is reflective of the housing system in most rural parts of Ghana, which was created with family structure in mind [52,53]. Although each wife might be in their individual room, privacy is almost nonexistent since the rooms are closer to one another and people share lots of common spaces.

Given that having several children is something to be proud of, women who choose to use contraception do so at a great risk of interpersonal conflict or social rejection [54]. According to Songsory [55] and Songsory et al. [56], the Northen region experiences the highest rates of poverty, which is often attributed to the prevalence of limited or nonexistent education among most residents. The availability of healthcare services is restricted due to the considerable distance between most health facilities and rural villages or community settlements [[7], [57]]. The insufficiency of qualified birth attendants and the limited accessibility to healthcare services, particularly for pregnant women, have been attributed to the lack of proximity to healthcare facilities. It is important to consider culture in this study because it has been expressed as a “psychic fabric, a second skin and an organic component of a nation” [58] which can impact the life and well-being of individuals.

Given this highlight of cultural context, the concept of revealing or concealing, expressing privacy boundaries can look different across cultures [[59], [60], [61], [62]]. Therefore, since studies on OF have not yet given attention to this added burden on women living with this condition, it will be beneficial to initiate scholarly conversations and studies on how women living with OF manage their private information. To achieve this goal, the study set out to answer the following research questions.

### 2.4 Research questions

RQ1a: How do women living with Obstetric Fistula (OF) manage disclosure practices or strategies after their diagnosis?

RQ 1 b: How do women living with Obstetric Fistula (OF) navigate non-disclosure practices/strategies after their diagnosis?

## 3 Method

### 3.1 Study design

To be able to answer the research questions guiding this study, the researchers conducted an interpretivist study using the social constructivism paradigm [63]. This method is the best fit since the researchers are positioning this study with the stance that meaning is socially constructed by people, their culture, and their contexts [64].

### 3.2 Considerations for feasibility, suitability, compatibility and yield

Our scholarly curiosity regarding this condition was piqued when we had an encounter with a woman who thought of giving up on life because of this condition. This interest guided our inquiry about feasibility, suitability, compatibility, and yield to the topic of [65]. To ensure that participants have the requisite experience, the inclusion criteria used for recruitment were specific to women who have lived with Obstetric Fistula (OF) or are still living with the OF condition for more than one month, whether they have had surgery for repair or not. It could be self-reported based on the symptoms associated with the condition or medically diagnosed. Interviewees must be 18 years of age or older and living in Ghana to give enough room for detailed stories and diverse experiences with the disease. To ensure feasibility, nineteen participants were recruited for the interview with consideration for data saturation [66]. Given that the participants in our study are considered “hidden" and challenging to access due to the social stigma associated with the OF condition [67], the researchers employed the snowball sampling technique to engage individuals with the condition who could facilitate connections with others willing to disclose their experiences. This approach resulted in a substantial volume of data.

### 3.3 Sampling technique and sample size

After approval from the Institutional Board Review, access to the participants was gained through researchers’ personal contacts. The purposive sampling technique was used first to ensure that participants met the inclusion criteria of identifying as a woman who has been medically diagnosed or self-report based on the symptoms of suffering from OF. Additionally, they were 18 years or above and resided in Ghana [68,69]. This study was limited to participants 18 years and above due to certain procedural constraints from IRB and difficulties in obtaining consent from parents of participants under 18 years old. Then snowball sampling was adopted to reach more participants, given that Lindolf and Taylor [70] as well as Tracy [65], posit that it is an effective way to reach difficult to access samples like OF patients. Participants who agreed were contacted to arrange a time when confidentiality, convenience, and comfort could be achieved. Participants were assigned pseudonyms to aid avoidance of any identifiers back to them. All participants ranged in age from 21 to 52 (M = 34.9, SD = 6.93). Most of the women were living in the northern part with a few numbers from the southern part of Ghana. There were 19 participants in total recruited for the interview which yielded 142 single-spaced pages of transcripts. The total amount of time spent on interviews was 20 h 13 min.

### 3.4 Data collection

The researchers employed a semi-structured interview protocol because it allows for unplanned additional questions as follow-up and can enable the study of a phenomenon that cannot be observed [71,72], which in this study is the disclosure/non-disclosure of. Interviews were conducted via phone call. The interviews were held via phone calls with participants mostly located in the Northern Region, Greater Accra, Western Region and Central Region. We used phone calls because in Ghana phone calls were affordable and the easiest way to communicate with those participants. Vogl [73] has suggested that there is more disclosure as participants are able to speak freely due to the sensitive nature of the conversation. Similarly, this aided in giving greater control to the interviewee to direct the conversation towards areas they perceive as important [74]. Aside from demographic information that was collected, interview questions were categorized to reflect the research questions and to elicit participants to narrate their experiences. Examples of some of the questions in the interview protocol are: “Did you share the information about your condition with anybody?“, If you did, who was it or who were they? And if you didn't? How did you come to make that decision? Interviews ranged between 40 and 60 min for each of the 19 participants. The study period was from April to July 2023. Data saturation was reached at the 19th interview. Data collection was stopped at this point since the participant's answers were very similar to those of the 18 people who had been interviewed before [6]. Compensation worth $20 was given to participants. A report by Ghana Health Services [5] revealed that the highest number of women living with OF come from the northern part of Ghana and this reflected as most of the participants came from the northern part of Ghana.

These were audio recorded, translated (those that were not in the English language) and transcribed by the researchers with consultation for verification of meaning from colleagues who were experts in the various languages. Colleagues who assisted in cross checking translation and transcribing data signed confidentiality statements to ensure data privacy [6].

The research team members are fluent speakers of the Ghanaian languages that participants used, therefore data accuracy were ensured, recordings of interviews were shared and assigned to each researcher to translate and transcribe, and then the same audio was given to another researcher to crosscheck to make sure that the meaning of the responses was captured as close to the English version as possible [75]. In cases where there are inconsistencies, another team member another team member reviewed the transcript to help reach a consensus. This helped to ensure less data loss in the process of translation and transcription.

Our translation decisions are influenced by the premise of staying as close as possible to the original meaning and accurately reproducing the interviewees’ statements in terms of validity. Due to limited project resources, engaging third-party translators was not feasible [76]. Out of the 19 interviews conducted, 3 were done in English while the rest were all in Ghanaian local languages (Twi-5, Fanti-2 and Dagbani-9).

### 3.5 Data analysis

To analyze the data, the researchers employed Tracy's [65] Phronetic iterative approach which allows for a concurrent process of data collection and data analysis. A “phronetic iterative approach alternates between considering existing theories and research questions on the one hand and emergent qualitative data on the other” [65]. This approach is appropriate for this study since the researchers started from a broader perspective and then narrowed the focus through abductive reasoning. This approach enhanced the researchers' flexibility to go “back and forth” into the data, literature, goals and theories as well as the researchers' knowledge to arrive at findings [65]. After translating and transcribing, we got about 142 pages of transcripts which were cleaned by checking for a closer to accurate transcription devoid of identifiers traceable to participants, repeated phrases, and redundant vocal fillers. We then read the transcripts multiple times for initial code generation which facilitated a sense of deeper understanding and closeness to the data.

During the first cycle coding stage, we adopted simultaneous coding [77,78], where we engaged in both descriptive coding [77,79], and in vivo coding [80,81] methods of analysis. These two coding methods helped us to describe to “readers to see and hear what the researchers saw and heard” [78] while maintaining what is significant to the participants in their own words [78]. The constant comparative method provided the avenue for checking in with our data for similar, new, or contrasting codes [63]. Codes generated were taken through the second cycle coding with the aim of reorganizing to search for and develop themes. Specifically, analytic coding and pattern coding [65], methods guided this phase of the study. Within the second cycle coding, the researchers approached the data deductively comparing and contrasting the strategies or practices identified already in the literature and also from CPM. This secondary cycle streamlined the categories to make analytical sense [65,78].

### 3.6 Qualitative trustworthiness

Several steps were taken to ensure high methodological rigor (Creswell, 2003). To achieve qualitative rigor, the researchers used thick descriptions to show the contextual meanings as it pertains to women living with OF in Ghana through the provision of details about the process, people, and activities told in their narratives [65]. Participants were asked probing questions that will encourage them to provide as many details as possible with the aim to aid us to “show” more than “tell” [65]. Additionally, peer review was conducted at different stages of the study allowing for other perspectives as well as opening up the study for additional scrutiny [82]. Lastly, we ensured heightened trustworthiness through the practice of member checking. Each participant was given the opportunity to review their respective interview transcript (reading to them) and encouraged to provide clarifications or suggest any necessary changes in case of potential misunderstandings. Additionally, in accordance with Maxwell's [83] recommendations, we increased trustworthiness by acknowledging our identities and positionalities. Recognizing and addressing researcher bias is crucial for maintaining credibility in qualitative research. To recognize and address researcher bias, the research team maintained a reflexivity journal to continuously monitor and challenge any assumptions of bias throughout the research process. Merriam et al. [82] also underscores the importance of researchers understanding and acknowledging their own identities and positions in enhancing trustworthiness. The significant contribution of the study is a key characteristic to achieve qualitative quality based on the implications highlighted [65].

In order to attain multivocality, the researchers recruited participants from diverse regions in Ghana who possessed varying backgrounds and perspectives. The aforementioned approach facilitated the acquisition of diverse perspectives and experiences, thereby enabling an in-depth understanding of women living with OF. This approach was implemented to prevent the limitation of findings to a singular perspective and to ensure that the intricacies and subtleties of the subject matter were sufficiently portrayed.

### 3.7 Researchers’ reflexivity and positionality

Reflexivity as a critical component of qualitative research has been emphasized as an important process that researchers must engage in to analytically reflect on one's own subjective assumptions which can influence the research encounter. In particular, our identity as Ghanaians who have been residing in Ghana for many years is noteworthy. As researchers, we are privileged to have some team members living in the same geographical area or region as the participants, while others are situated outside the region. Our different locations allow us to gain both an insider and outsider perspective on this research. We position ourselves as an insiders-outsiders which Merriam et al. [84] have indicated that both positions have their challenges and benefits. As insiders, we are privy to most of the cultural underpinnings as we have stayed all our lives in Ghana, three of our team members are women, one member has gone through the labor process four times and knows how it feels and can relate with the process. This insider position provided us with the foundation to connect perceptual ideas to “real” experiences to form a holistic interpretation. Richardson and St Pierre [85] asserted that the “product cannot be separated from the producer, the mode of production, or the method of knowing” (p.476). On the other hand, as outsiders, we engaged with our participants as researchers with the goal of gathering data to find answers to the research questions [65]. Therefore, we are reflexive of the above biases and how they may have influenced the research design, data collection and data analysis.

## 4 Ethical considerations

This study adheres to the principles outlined in the Declaration of Helsinki and has received approval from the Institutional Review Board (IRB) of Arizona State University. Ethical considerations were paramount throughout all stages of the research process, from study design to data collection, analysis, and dissemination.

All participants received detailed information about the research purpose, procedures, risks, and benefits of the research prior to their participation in the study. We obtained informed consent from each participant, ensuring they were fully aware of their rights, including the voluntary nature of their involvement and the option to withdraw at any time without penalty. Before proceeding with the interview, we read consent forms in the participants' preferred language and addressed any questions or concerns they raised. This study opted for verbal informed consent. This decision was informed by two key considerations. Firstly, the research team recognized that a significant portion of the target population possessed limited literacy skills. Utilizing a written consent form could have inadvertently excluded these individuals, thereby hindering the study's inclusivity. Employing verbal consent ensured that all women, regardless of their literacy level, had an equal opportunity to participate and contribute their valuable experiences. Secondly, the study design itself presented logistical challenges related to written informed consent. The targeted population lacked access to reliable communication channels, such as email, which would have been necessary for disseminating a written consent form. Furthermore, the phone interview format, coupled with the potentially stigmatized nature of the research topic, could have induced anxiety in participants. Verbal consent, delivered in a clear and comprehensive manner, facilitated a more conversational approach to informed consent, potentially mitigating apprehension associated with the formal nature of a written document. We used pseudonyms throughout the study to protect participants' privacy and confidentiality. To maintain anonymity, we removed or altered any identifying information that could potentially reveal participants' identities. Only members of the research team had secure access to the data collected during interviews and focus group discussions. We strictly followed confidentiality protocols to prevent unauthorized access to sensitive information.

We took special care to minimize any potential harm or discomfort participants may experience during the research process. Sensitivity to the sensitive nature of obstetric fistulas and their impact on women's lives guided the design of interview questions and the approach to data collection. We assured participants of non-judgmental and supportive interactions and encouraged them to share their experiences at their own pace and comfort level. Participants who expressed a need for additional assistance received referrals to support services and resources. This study is driven by a commitment to promoting the well-being and empowerment of women living with obstetric fistulas. We aim to raise awareness, stimulate dialogue, and inform policy and practice interventions aimed at improving women's quality of life by amplifying their voices and experiences. Findings from this research may contribute to the development of more responsive and culturally sensitive healthcare services, as well as advocacy efforts to address the underlying determinants of obstetric fistulas and reduce their prevalence.

## 5 Findings

Guided by the CPM, this study explored disclosure and non-disclosure practices that OF patients adopt in managing their private information after diagnosis. In answering the research questions, the analysis yielded themes that were first separated under disclosure/non-disclosure practices broadly from the data reduction, the following categories were highlighted: recipients/who received the disclosure, when the disclosure happened, how much/little was shared (breadth and depth/content) and specific modes used to reveal or conceal the information. Each of these categories generated specific predominant practices that participants used in either revealing or concealing the diagnosis of the OF condition. The terms strategies and practices were used synonymously due to their usage in disclosure literature. This section presents examples from the interviews that demonstrate the themes across both disclosure and non-disclosure strategies adopted by participants.

### 5.1 Women living with of disclosure strategies

Findings show practices used by participants to manage their OF diagnosis information while they decided to make other co-owners. The four broad categories of who, when, what and how the information of their diagnosis became known to others were each discussed with specific nine communicative practices participants adopted. This extends not only to the initial diagnosis but throughout the condition till recovery. See Tab 2 for highlights of summary findings.

### 5.2 Who receives the disclosure?

In response to who receives the disclosure, participants described three categories of people who became co-owners of the disclosure which the researchers classified as primary co-owners, secondary co-owners, and accidental co-owners.

The primary co-owners were the group of people who were either partners or immediate family members. Participants shared some closeness or bond with them, so they indicated that such people had the right to know. These people were mostly husbands or parents of the participants. For instance, the following phrases were common in the responses from participants: “I informed my husband and mother first …“; “So I told my mum immediately …” and “My husband is the only one I told”. The use of these words in their statements emphasizes those whom they “first”, “only” and “immediately” decided needed to receive information about their diagnosis.

The second group was the secondary co-owners who were made up of some selective individuals whose relationship with the participant was based on either past experience with them, closeness to or the need for support from them. These were mostly siblings, in-laws, close friends, religious leaders, or some extended family members. For example, Ekua said, “… my mother-in-law also knew about it because of the relationship I had with her …” while Efua explained, “I told my pastor, so the church initially helped me …“. Most of these recipients as shown were added co-owners whom participants decided they should tell to strengthen the relationship or get some support from them.

The accidental co-owners included neighbors, some extended families, co-workers, or friends. One of the participants, Yaa, established that the neighbors in their house became co-owners accidently through conflict when she used to quarrel with her husband. Yaa clarified:

For those in my house they got to know because whenever I quarrel with my husband, it comes up and he will use that to insult me, so they heard.

Here, Yaa established that the neighbors in their house became co-owners accidently through the conflicts she used to have with her husband. In these cases, participants added that it was as a result of the nature of residence/housing (“compound houses” or small villages) they lived in.

### 5.3 When do we disclose?

The “when” of the disclosure means at what point in relation to the diagnosis did participants share the information with others. Two practices emerged: spontaneous and procrastinated disclosure (“at the right time”).

For most participants, the spontaneous strategy was used for the primary and secondary co-owners. The spontaneous practice was adopted in instances where participants were unsure and became afraid when they noticed the symptoms or became worried after being diagnosed by the healthcare practitioner. Afi, a farmer who was staying with her husband depicted this by saying, “When I was discharged, I started experiencing pains and other symptoms therefore complained to my husband immediately …“. Afi in the above excerpt admitted telling the partner instantly due to the uncertainty she felt at that moment.

However, there were a few occasions where disclosure of the diagnosis was delayed because participants considered waiting to disclose the diagnosis at “the right time”. The notion of procrastinated disclosure happened when participants lacked communication efficacy or wanted to gain the courage to share the secret of the diagnosis. For example, Araba, who became divorced after the condition and is still living with the condition recounted:

… I only told my mother-in-law after a while because I did not know how to say it to her and I was looking for the right time, I think but later I had too because she is the only one who will help.

Araba demonstrates the tension she experienced while finding the “right words” to use to share her diagnosis with the mother-in-law. In managing this disclosure, she weighed the risk-benefit and concluded that she needed support, which she believed was the right time in her case.

### 5.4 What do we disclose?

Concerning what to disclose, participants talked about how much or little they shared with others about their condition. Thus, emphasizing the amount of content that the discloser wanted others to know as their way of managing their private information. From the data, these were classified into full and partial disclosures.

For the full disclosure, participants commented that they explained in detail all the symptoms to the confidants who were mostly partners or close family members and friends whom they trusted with their secret or knew they could offer them support. Baaba mentioned that “I told my husband, then my best friend who was a nurse, everything happening in my body because it was strange and not easy”. She continued and said, “I couldn't take it alone anymore”. Expressing desperation, Baaba divulged to her husband and best friend the symptoms of the condition since the condition made her uneasy and she needed to seek help. On the other hand, Amina talked about how she managed her disclosure to the in-laws, saying:

I told them I am sick and need an urgent operation, but I did not give them details, these people if you tell them everything then everyone in the family will know, then they can even say they will not eat your food or drink your water.

This participant who was living with her husband and her in-laws in the same compound explained how she partly revealed to the in-laws about her diagnosis to avoid the stigma and avoidance that may result from disclosing her diagnosis. This was her way of taking control and setting the boundary so that information about her diagnosis did not spread to others.

### 5.5 How do we disclose?

The “how” focuses on specific modes women living with OF adopted in managing the revealing of their private information to others. Two main practices emerged from the data: The Direct and Third-Party Modes [40].

***Direct disclosure.*** To explain the direct mode, participants described instances where they initiate the revelation to others by themselves in a face-to-face/telephone call interaction or they told others directly as a response to external forces who pressured them to know. The face-to-face could be alluded to by the proximity of most of the confidants who received the disclosure. All participants who were living in rural/remote areas in Ghana used the face-to-face direct method because confidants were closer to them in relation to distance. Whereas the few who mentioned the direct telephone call method lived in the cities and had some of their families staying in different communities. One participant responded, “I called my mother on the phone and told her because I could not travel there”. Most of the participants in this study admitted to using this strategy since it was convenient and culturally appropriate. Also, breaking negative news like their diagnosis needs to be done in person if possible.

***Third-party disclosure***. The third-party disclosure mode was used sparingly in this study. This is where the patients themselves were not the source of the disclosure but there was the presence of another person who revealed it on behalf of the participants. In this strategy, participants characterized two distinct forms of disclosure: expert third-party disclosure and non-agentic third-party disclosure. First, the expert third-party disclosure happens through somebody who has the expertise or status which in this study were healthcare workers to share the information with others. For example, Muniratu mentioned that “The doctor told us together at the hospital”. Apparent in this statement is the evidence that some of the co-owners were present as caregivers with the participants when the healthcare worker confirmed the diagnosis as fistula, making those third-party individuals aware of the participant's condition. Second, the non-agentic third-party disclosure considered instances where there is a third-party exposure of the diagnosis without explicit consent from the women living with the OF. This takes away agency and control from the patients. Most of these non-agentic third-party who disclosed information were family members who became earlier confidants in the diagnosis and then passed on the information to others. Illustrating this from the data, Akos indicated:

The doctors informed my husband because he was with me at the hospital, and my husband informed my relatives. The reason was also that I needed a blood transfusion ….

Here, Akos gave a clear example of how these two forms of third-party disclosure unfolded. So, within the expert third party, the providers disclosed the information to the patient while her husband was present. Subsequently, in the non-agentic third-party, the husband as a co-owner of the information, then took control of the information and shared it with others to get support from them.

### 5.6 Women living with of nondisclosure strategies

In the current study, non-disclosure or concealment are used interchangeably to mean participants did not tell the information about the diagnosis to people they did not want them to know. Thus, to manage the nondisclosure, they resorted to different practices to keep the information from others. Women living with the OF condition who made the decision not to disclose recounted either having disclosed before and got a negative reaction so learned their lessons or decided from the beginning never to tell anybody about the diagnosis till they recover or recovered (for participants who have had surgery already).

### 5.7 Who do we conceal from?

People with whom participants felt reluctant to disclose their diagnosis ranged from partners, close and distant family members, in-laws to cohabiting partners, friends, co-workers, and people classified as “outsiders.” The non-disclosure for some was permanent, so those people have not been told and may never know. One of these women expressed her decision stating that “I have not told the people in my new place that I have this condition, they will never know.” Another participant also stated that “I never want anybody to know until I recover; no, this is shameful.” These two women demonstrated their resolution never to tell others about their diagnosis as a way to manage the privacy of their information. They could not bear the negative reaction or risk their disclosure may have on them. Nonetheless, there was the temporary non-disclosure where the diagnosis was concealed for a short time before they were later revealed. I such instances, revealing the diagnosis was mostly with the intent of seeking support from others. One of the participants, Barikisu, indicated her intent to reveal her diagnosis to her uncle.

I will be telling my uncle soon about my condition because now I do not have any money for treatment, I have spent everything on this disease.

Barikisu planned to disclose to the uncle later. While she indicated that she was not exactly sure when she would disclose the information to her uncle, she was definite about her current decision to reveal the diagnosis to her uncle for help, given that she was financially burdened and had no hope for repair.

### 5.8 How did we conceal?

Participants outlined certain modes they use or used to conceal their diagnosis from others. Three themes emerged from these communicative practices: Active avoidance, Active pretense, and Active involvement.

***Active avoidance***. One of the prominent practices described was active avoidance which explains how participants make the effort of not mingling with or being in the midst of people who can probably ask about their condition or even suspect them of being sick. Therefore, they avoided gatherings or people in general who they do not want to know about their diagnosis. One participant confirmed this by saying that:

Yes, I am always indoors. I only come out when I realize people are not outside. When they come out, I quickly run indoors. I am staying in a compound house, so I have to be careful because they already like to gossip.

Within this example, the woman had to actively distance herself from the neighbors who were living with her in this type of house with the hope of not being spotted by any of these “gossips” as she calls them. Such active avoidance, she believed, would allow her to keep her condition a secret.

***Active pretense***. Again, other OF patients had to actively feign to others that they are doing well to avoid the negativity attached to the condition. To achieve this pretense and make it believable, participants expressed that they engaged in using excessive and dangerous items such as “polythene bags”, “cloth”, “glue” and others to keep them from soiling themselves with urine or feces. This respondent crying through this lamented, Before I realize, they all will go and tell people about my condition, and they will be laughing at me. So, where I am staying currently, I have not told people that I have this condition. I pretend I am okay, but I am not because I don't want people to know about my condition.

Narrating this story further, she explained, “Hmmm, I make sure I have a polythene bag, glue and something on me to stop the leakage. Even in pain, I keep it under control and say I am okay though I am not. It is a lot … (Crying). In this excerpt, this woman would go to any extent to conceal her secret because of her previous experience with stigmatization in her previous place of residence. Others in similar situations could use several of these dangerous items to feign being healthy and accepted by others.

***Active involvement*.** In contrast to active avoidance is active involvement. Participants using this strategy illustrated how “they put themselves out” by engaging in most of the activities and gatherings so that people will not have the cause to “suspect” them of any sickness or being forced to share with people their diagnosis. Therefore, most of these women still struggle through the pain and weakness to continue their jobs or attend events to keep people from asking questions. Consistent hygienic practices as a strategy to conceal the secret of their condition were used often with either pretense or active involvement. Participants expressed how they were intentional and cautious about cleaning themselves as frequently as possible along with wearing pads and changing them consistently to reduce the smell or leakage showing on them. Some of these hygienic practices included frequently washing themselves, wearing a pad and as they changed frequently and dressing neatly. All of these were done with the intention of concealing from people their condition. Abena recounted,

… Every day, I used to take good care of myself and pad it, then when I realized that it was getting worse, I would go back and change it. I didn’t leave myself to the extent that people will know I was having such a condition or suffering from anything.

For Abena, this was a daily battle for her to conquer and as frustrating as it may seem she is not ready to leave it to chance for her secret to be out there.

The strategies used by these women suffering from OF either to conceal or reveal were all practices to maintain control and set boundaries to manage their private information. Participants altogether acknowledged that making the decision and engaging in these practices did not come about unexpectedly but they had to negotiate several dialectic tensions established in addressing this information sharing [26,37].

## 6 Discussion

The purpose of this research was to understand how women living with OF manage disclosure/non-disclosure practices after their diagnosis. Specifically, this was explored in relation to the broader categories of co-owners of disclosure/non-disclosure, when the disclosure occurs, how much of the content is shared or not and the medium or modes used in delivering or concealing the information about their diagnosis to others. Using CPM, this section analyzes three key significant contributions this paper makes pertaining to information privacy management and the communicative practices of women living with OF. Hence, these findings have significant contributions to theory and practical application.

Findings revealed the dialectic pull and push involved in making decisions about communicative practices that can be useful for revealing or concealing one's private information. Similar to other previous findings from Venetis et al. [43] and Afifi and Steuber [40], the study significantly promotes revealing and concealing not to be seen as a self-event or one-person act but can also advance towards a social process. This opens further discussions, especially for collectivist cultures that situate information about one family member as a piece of the group information that is guided by a collective boundary for managing it [26]. Ownership among OF patients in this study with their spouses is perceived as collective ownership [44] so both have the same right to manage the information further without explicit boundaries set. This also reflects the Ghanaian patriarchal society's view of the headship of the husband in marriage [52]. This tends to stretch the concept of ownership in CPM further. However, the criticality of choice as studied by Petronio [26] in CPM was absent among OF patients due to their marital status, financial power. gender, and stigmatized conditions.

In addition, the current study provides an empirical example of how disclosure or non-disclosure involves a series of complex processes and not a one-time event. This is evident from the current findings that show that although previous studies found women to be the ones likely to disclose more than men [31], most OF patients lacked the efficacy to communicate their disclosure especially when they perceive the risk as enormous. Interestingly, most participants agreed that they sometimes engage in both disclosures at one point for some people while at the same time engaging in non-disclosure for other selective individuals. Although CPM asserts privacy management involving disclosure and privacy as distinct opposing tensions, Omarzu [86] gave a contrasting view that disclosure and non-disclosure must not be seen as two opposite events that happen one time but recognized as a social process that can change over time. Confirming this Bute and Vik [48]“conceptualizing privacy management as a process not only advances current theoretical understandings but also has implications for individuals who are coping” (p.2). OF women's responses from the findings showed that the decision to disclose or not disclose evolves over time therefore they tend to shift privacy boundaries as the situation demands. This adjustment is made to either increase or decrease their permeability in response to pressures from given situations [87], thereby emphasizing the “contextual criteria” [26].

Finally, it is important to point out that most of the non-disclosure practices were unique due to the type of disease and cultural context [24]. For instance, most of the practices for concealing (e.g., active avoidance, active pretense, and active involvement) adopted by these women can be dangerous to their well-being and end up worsening their health condition. Most participants expressed using unhygienic materials to conceal their secrets, while others worked through pain and discomfort to achieve the same goal of keeping their secrets away from others. Also, the non-agentic third-party disclosure looks different from the studies that identified third parties as a strategy [40,42]. The control or agency is taken away from the patient, which oversteps the boundary rule provided in CPM [26].

## 7 Implications

This study outlines two key implications pertaining to theoretical and practical implications. Considering the theoretical implications, given that CPM describes privacy as sustainable through the privacy management system process in a real-life context, this study contributes to a better understanding of how women living with OF navigate their private information when they are making decisions to reveal or conceal it. In general, a non-Western perspective is provided to exemplify not only a different way of control and boundary setting in a predominantly collectivist culture but also to consider disclosure or non-disclosure as an ongoing engagement where people can reframe or change earlier exchanges.

On practical implication, engaging in this study gives a practical outlook to stakeholders and policymakers in developing countries to provide interventions that will guide women in these conditions through the complex process of disclosure or non-disclosure. This intervention will provide relief for women who are living with OF as they are already pressed with multifaceted layers of effects. Therefore, presenting alternative strategies through programs along with practical conversation can help increase their efficacy after diagnosis.

## 8 Limitations and future research

Given the unique context and scope of the study, care should be taken when applying the findings to other contexts. The constraint may be found in relation to culture, gender, sexual orientation, and type of condition. Participants identified as cisgender and were in a heterosexual relationship with their partners. However, most of the findings discussed in this current paper may be transferred to other similar contexts. Another limitation hinges on language pertaining to translating and transcribing procedures. Since a majority of the participants were fluent in the Ghanaian local languages, participants who spoke local languages that were not spoken by the researchers were excluded from the recruitment, which privileges the views of certain ethnic groups over others in the study. Relatedly, translating some words from the local language to English was challenging since translating can distort the actual meaning of some words. Future research could extend and go in-depth by including how these practices impact their romantic, family, or friendship relationships.

## 9 Conclusion

Overall, this study provides initial insights into the understudied issue of revealing or concealing strategies adopted by women living with Obstetric Fistula through the lens of Communication Privacy Management Theory. The decision to disclose or not has been established as a complex process which happens not as a one-time event but changes over time. Therefore, women living with Obstetric Fistula are coupled with the added burden of who, when, what and how they manage their diagnosis information. Providing interventions geared toward disclosure and non-disclosure strategies after diagnosis can provide enormous relief for these women.

## Ethics declarations

This study was reviewed and approved by Arizona State University Institutional Review Board (IRB) with the approval number STUDY00017496. This study did not require clearance from any institution in Ghana since the hospitals were not contacted for any assistance in reaching the participants for the study. Hence, the participants interviewed were not affiliated with any institutions but the general population who gave consent to be interviewed. This study complies with all regulations. All participants/patients provided informed consent to participate in the study.

## Data availability

The data will be made available from the corresponding author upon reasonable request.

## CRediT authorship contribution statement

**Sandra Freda Wood:** Writing – review & editing, Writing – original draft, Visualization, Validation, Project administration, Methodology, Investigation, Funding acquisition, Formal analysis, Data curation, Conceptualization. **Ebenezer Ato Kwamena Aidoo:** Writing – review & editing, Writing – original draft, Visualization, Validation, Supervision, Software, Resources, Project administration, Methodology, Investigation, Funding acquisition, Formal analysis, Conceptualization. **Kweku Ewusie Orleans Lindsay:** Writing – review & editing, Writing – original draft, Methodology, Investigation, Formal analysis. **Jessica Afful Tuleassi:** Writing – review & editing, Writing – original draft, Project administration, Investigation. **Frank Nukunu:** Writing – review & editing, Writing – original draft, Supervision, Methodology. **Cindy Afoakwa-Acheampong:** Writing – review & editing, Writing – original draft.

## Declaration of competing interest

The authors declare the following financial interests/personal relationships which may be considered as potential competing interests: Participants where only given compensation of $20. The funds was acquired by first Author from Transformation project- Hugh down school of human communication.

## Figures and Tables

**Table 1:** Demographic Characteristics of Participants

Characteristics	Number of Participants (n = 19)	Percentage %
Age range (years)		
Not sure	1	5.2
21–30	6	31.6
31–40	9	47.4
41–50	2	10.5
51–60	1	5.3
Resident Region		
Upper East Region	2	10.4
Upper West Region	3	15.8
Northern Region	9	47.4
Western Region	2	10.5
Ahafo Region	1	5.3
Central Region	1	5.3
Ashanti Region	1	5.3
Education Level		
Nil	8	42.1
Primary	2	10.5
Junior High	3	15.8
Senior high	3	15.8
Vocational	1	10.5
Tertiary	2	5.3
Marital Status		
Single	1	5.3
Married	14	73.7
Cohabiting	2	10.5
Divorced	2	10.5
Number of Children		
0–2	9	47.4
3–5	8	42.1
6–8	2	10.5
Religion		
Muslim	14	73.7
Christian	5	26.3
Health Insurance		
Yes	19	100
No	0	0
Occupation		
Trader	7	36.8
Farmer	5	26.3
Skill based employment	3	15.8
Fishmonger	1	5.3
Government organization	1	5.3
Private organization	2	10.5
Duration of Condition		
1–5 years	14	73.7
6–10 years	2	10.5
11–15 years	2	10.5
16–20 years	1	5.3
Repair Done		
Yes	13	68.4
No	6	31.6
Baby Survived		
Yes	12	63.3
No	6	31.3
Not Applicable	1	5.4
Hospital Facility Usually Visited		
Public Hospital	11	57.9
Private Hospital	1	5.3
Community Health Center/clinics	7	36.8

*Note.* This table shows the demographic information of all participants in this study*(n* = 19) for about 20 h of interviews yielding 142 single spaced pages of transcription.

**Table 2:** Summary highlights of findings

Disclosure strategies/practices
WHO
▪ Primary Co-owners
▪ Secondary Co-owners
▪ Unaware Co-owners
WHEN
▪ Spontaneous
▪ Procrastinated disclosure (at the right time)
WHAT
▪ Full revelation
▪ Partial revelation
HOW
▪ Direct
● Face to face
● Telephone
▪ Third Party
● Expert third party
● Non-agentic third party
Non- disclosure strategies/practices
WHO
Selective
HOW
▪ Active Avoidance
▪ Active Pretense
▪ Active Involvement
